# Effects of Different pH Control Strategies on Microalgae Cultivation and Nutrient Removal from Anaerobic Digestion Effluent

**DOI:** 10.3390/microorganisms10020357

**Published:** 2022-02-03

**Authors:** Hyeonjung Yu, Jaai Kim, Chaeyoung Rhee, Juhee Shin, Seung Gu Shin, Changsoo Lee

**Affiliations:** 1 Department of Urban and Environmental Engineering, Ulsan National Institute of Science and Technology (UNIST), 50 UNIST-gil, Eonyang-eup, Ulju-gun, Ulsan 44919, Korea; yougusgus@unist.ac.kr (H.Y.); jaai@unist.ac.kr (J.K.); 2 Department of Energy Engineering, Future Convergence Technology Research Institute, Gyeongsang National University, 501 Jinju-daero, Jinju 52828, Gyeongnam, Korea; chaeyoung415@gnu.ac.kr (C.R.); shinjh@gnu.ac.kr (J.S.); sgshin@gnu.ac.kr (S.G.S.)

**Keywords:** anaerobic digestion effluent, ammonia loss, mixed-culture microalgae cultivation, *Parachlorella*, pH control

## Abstract

This study investigated nutrient removal from anaerobic digestion effluent by cultivating mixed-culture microalgae enriched from anaerobic sludge under different pH conditions: R_UC_ (uncontrolled), R_7__–8_ (maintained at 7–8), and R_<8_ (maintained below 8). Significant amounts of NH_4_^+^-N were lost by volatilization in R_UC_ cultures due to increased pH values (≤8.6) during the early period of cultivation. The pH control strategies significantly affected the biological NH_4_^+^-N removal (highest in R_7__–8_), microalgal growth (highest in R_7__–8_), biomass settleability (highest in R_<8_), and microalgal growth relative to bacteria (highest in R_<8_) in the cultures. *Parachlorella* completely dominated the microalgal communities in the inoculum and all of the cultures, and grew well at highly acidic pH (<3) induced by culture acidification with microalgal growth. Microalgae-associated bacterial community structure developed very differently among the cultures. The findings call for more attention to the influence and control of pH changes during cultivation in microalgal treatment of anaerobic digestion effluent.

## 1. Introduction

Microalgae have gained increasing attention in recent decades because of their promising potential for wastewater treatment, CO_2_ mitigation, as well as biofuel, biochemical, and biomaterial production [1,2]. Microalgae grow photosynthetically using CO_2_ as a carbon source while assimilating nutrients (i.e., N and P) in wastewater and accumulating organic compounds intracellularly. Cultivating microalgae with wastewater can minimize the consumption of water and nutrients (supplied as chemical salts), the high cost of which is a major challenge for large-scale microalgae cultivation [3]. Accordingly, extensive studies have been conducted on the cultivation of microalgae using different wastewaters for the dual purposes of producing microalgal biomass for biorefineries and treating wastewater [1,2]. The growing global shortage of water and resources highlights the benefits of wastewater-based microalgae cultivation for sustainability, although more research is needed for practical application, especially regarding long-term stability [3].

The conversion of organic wastes into methane-rich biogas through anaerobic digestion (AD) is increasingly recognized as a viable option for sustainable energy production. Although AD is a mature technology that has long been and is increasingly used worldwide to treat various wastes, there still are some challenging issues limiting its wider application. Foremost among them is the high concentration of ammonia nitrogen in the effluent, which needs to be further treated before discharge to prevent nutrient pollution and eutrophication of water bodies [4]. Ammonia nitrogen removal from wastewater is most often performed by conventional nitrification-denitrification processes, such as the A2O process, which are effective but require energy-intensive aeration and external organic carbon addition. These drawbacks reduce the economic efficiency of the processes significantly, especially when treating high-ammonia wastewater with low levels of bioavailable organic carbon such as AD effluents [5]. For more cost-effective treatments, several alternative ammonia nitrogen removal technologies, for example, by different anammox-based processes, simultaneous and shortcut nitrification-denitrification processes, bioelectrochemical systems, and microalgae cultivation, have been extensively explored and applied in recent years [6].

Effective nutrient removal and recovery from AD effluent have been demonstrated using various microalgal species and mixed consortia of microalgae and bacteria [4,7]. Cultivated microalgal biomass during the treatment can be used as a feedstock for biofuels, biochemicals, animal feed, fertilizer, or other bioproducts, or fed to the digester as a co-substrate to increase biogas production, which can improve the economic and environmental benefits of AD [7,8]. AD effluents generally contain several thousand milligrams per liter of total ammonia nitrogen (TAN) and are mostly used after dilution to avoid ammonia inhibition of microalgal growth [4,7]. Meanwhile, there can be insufficient nutrients if the medium is too dilute, and therefore, proper dilution of AD effluents is important for successful microalgae cultivation. Note that the fate of ammonia (pKa = 9.3 at 25 °C) is directly affected by the medium’s pH. The more alkaline the medium, the greater the fraction of TAN present in the form of toxic and volatile free ammonia (NH_3_). Therefore, a significant loss of ammonia by volatilization can occur in microalgae cultures (semi)continuously aerated for CO_2_ supply, depending on the medium’s pH. In such cases, the contribution of microalgae to the removal of ammonia is limited, in other words, microalgal growth is limited by nutrient availability. Such adverse effects are even more pronounced when using more dilute AD effluents with lower nutrient concentrations. Furthermore, microalgal growth can be inhibited due to NH_3_ toxicity at alkaline pH.

In addition to having direct effects on NH_3_/NH_4_^+^ equilibrium and nitrogen availability, pH is a crucial factor that determines the growth of microorganisms, including microalgae. Different microorganisms have different pH ranges for optimal growth, and changes in environmental pH influence the functional activity of microorganisms, especially the activation of enzymes and proteins [9]. Although the majority of microalgae are known to favor neutral pH, different microalgal species have been found to grow under highly acidic (pH < 5) or alkaline (pH > 9) conditions [10]. Meanwhile, microalgal growth can change the medium’s pH, which in turn affects the growth of microalgae. A study on the proton imbalance during microalgae cultivation reported that alkalinity was produced or consumed depending on the nitrogen source and its metabolic process, and that this had a greater effect on the medium’s pH than did CO_2_ assimilation [11].

Accordingly, it is important to understand how the medium’s pH influences the microalgal growth and utilization of ammonia nitrogen and whether pH control is beneficial for effective microalgal treatment of ammonia-rich AD effluents. However, little attention has been paid to the changes and effects of pH during microalgae cultivation in wastewater, which may lead to a significant misestimation of the capacity of nitrogen removal by microalgae. To address this gap, the present study compared the effects of different pH control strategies (i.e., no control, below pH 8, and between pH 7 and 8) on the nutrient removal and biomass production during the cultivation of a mixed microalgal consortium derived from anaerobic sludge in a series of diluted AD effluents. For deeper insights, the microbial community structures in the different experimental cultures were comparatively analyzed by high-throughput sequencing (HTS), in addition to physicochemical monitoring. This study provides useful information and the basis for further research on the pH changes and control measures in wastewater-based microalgae cultivation.

## 2. Materials and Methods

### 2.1. Microalgae Cultivation in AD Effluent

The mixed-culture microalgae cultivation experiments were divided into three groups with different pH control strategies: R_UC_ (uncontrolled) as a control, R_7__–8_ (controlled between pH 7 and 8) representing the optimal conditions for microalgal growth [9], and R_<8_ (kept below pH 8) set to avoid the volatilization loss of ammonia. Each strategy was tested under five different culture conditions in duplicate: four different initial NH_4_^+^-N concentrations (100, 200, 400, and 800 mg/L) and an uninoculated control condition at 800 mg NH_4_^+^-N/L (Table 1). The cultures were cultivated in batch mode in 550-mL Erlenmeyer flasks without baffles, with a working volume of 500 mL. Each flask was filled with 450 mL of AD effluent and inoculated with 50 mL of a subculture of a microalgal mixed culture previously enriched from the sludge of a lab-scale anaerobic digester treating *Ulva* slurry [5]. The AD effluent collected from a full-scale digester treating sewage sludge and food waste was centrifuged at 3400× *g* for 20 min, and the supernatant was used as the medium for microalgae cultivation after dilution with distilled water to the desired NH_4_^+^-N concentrations. The carbon and nitrogen concentrations in the microalgae inoculum, raw AD effluent, and initial culture mixtures (time 0) are shown in Table 1. A total of 30 flask cultures (i.e., 3 pH control strategies × 5 culture compositions × 2 replicates) were cultivated for 30 days at room temperature (25–27 °C) under white light-emitting diode illumination (3000 lx ) in a 16-/8-h light/dark cycle. Each flask was aerated via bottom bubbling with ambient air (170 mL/min, 0.34 L air/L culture·min) under constant magnetic stirring at 200 rpm. In the R_7__–8_ and R_<8_ cultures, the pH was adjusted daily as needed using 2 M HCl or NaOH solution.

### 2.2. Physicochemical Analyses

Chlorophyll concentration and optical density at 680 nm (OD_680_) were analyzed to monitor the growth of microalgal biomass during cultivation. Pigments were extracted from the pelleted biomass of each culture (12,000× *g*, 10 min) with dimethyl sulfoxide, and the concentrations of chlorophylls a and b (Cha and Chb) were estimated from the absorbance of the extracts at 649 nm (A_649_) and 665 nm (A_665_) using Equations (1) and (2) [12] (total chlorophyll (Cht) was determined as the sum of Cha and Chb) as follows:

Cha concentration (mg/L) = 12.47A_665_ − 3.62A_649_ (1)

Chb concentration (mg/L) = 25.06A_649_ − 6.5A_665_ (2)

Settleability of cultivated biomass was determined as previously described [13]. An 8 mL aliquot was taken from each microalgae culture on the last day of cultivation (Day 30) and settled by gravity in an 11 mL cylindrical glass vial (1 cm diameter, 14 cm height) after vigorous shaking. The decrease in OD_680_, as measured directly from the vial at 2 cm above the bottom, was monitored for 24 h at room temperature. The settling efficiency was calculated at 12 and 24 h using the following equation:

Settling efficiency (%) = {(OD_0_ − OD_t_)/OD_0_} × 100 (3)

where OD_0_ is the OD_680_ measured at time 0 and OD_t_ is the OD_680_ measured at time t.

Solids were measured according to Standards Methods for the Examination of Water and Wastewater [14]. Total nitrogen was analyzed using an HS-TN(CA)-H kit (HUMAS, Daejeon, Korea). Total and inorganic carbon contents were determined using a TOC-V CPH analyzer (Shimadzu, Kyoto, Japan). NH_4_^+^-N was measured using an ion chromatograph (Dionex ICS-1100, Thermo Scientific, Waltham, MA, USA) fitted with an IonPac CS12A column (Thermo Scientific, Waltham, MA, USA); NO_2_^−^-N, NO_3_^−^-N, and PO_4_^3−^-P were quantified using the same ion chromatograph fitted with an IonPac AS14 column (Thermo Scientific, Waltham, MA, USA). Samples for total and inorganic carbon analysis and for ion chromatography were filtered through a 0.22 μm pore syringe filter. All of the analyses described in this subsection were performed in duplicate for each sample from the duplicate microalgae cultures (*n* = 4), and the standard deviation of the four measurements was used to plot the error bars for each data point.

### 2.3. DNA Extraction and Digital Polymeric Chain Reaction

Total DNA was extracted from the inoculum, AD effluent, and culture samples taken at the end of the 30-day batch cultivation using an automated nucleic acid extractor (ExiProgen, Bioneer, Daejeon, Korea), as previously described [15]. The extracted DNA was eluted in 100 μL elution buffer and stored at −20 °C. The copy concentrations of bacterial 16S rRNA genes and eukaryotic 18S rRNA genes were analyzed by digital polymeric chain reaction (dPCR) using a bacteria-specific BAC primers/probe set [16] and a eukaryote-specific 528F/706R primer pair [17,18], respectively. The dPCR mixtures for quantifying bacteria and eukaryotes were prepared using a QIAcuity Probe PCR Kit and a QIAcuity EvaGreen PCR Kit (QIAGEN, Hilden, Germany), respectively, according to the manufacturer’s instructions. The prepared mixtures were loaded onto a QIAcuity Nanoplate 26 K 24-well (QIAGEN, Hilden, Germany), which could run 24 samples (40 μL/well) with up to 26,000 partitions (i.e., individual 0.91 nL reactions) per well. The dPCR reactions were amplified and analyzed in a QIAcuity ONE 2-Plex system (QIAGEN, Hilden, Germany) with the following thermal cycling conditions: an initial heat activation at 95 °C for 2 min and 40 amplification cycles (two-step cycling at 95 °C for 15 s and 60 °C for 30 s for bacterial amplification and three-step cycling at 95 °C for 15 s, 60 °C for 15 s, and 72 °C at 15 s for eukaryotic amplification), followed by cooling at 40 °C for 5 min for eukaryotic runs only. A non-template control was included in each dPCR run to set the signal-to-noise threshold. The number of target genes in a sample was determined from the fraction of positive partitions (i.e., amplified reactions with fluorescence signal) using Poisson statistics.

### 2.4. High-Throughput Sequencing

The microbial community structure was analyzed by high-throughput sequencing (HTS) in the inoculum, AD effluent, and selected microalgae cultures (i.e., all cultures at initial NH_4_^+^-N concentrations of 100 and 400 mg/L and the inoculated and uninoculated R_<8_ cultures at 800 mg NH_4_^+^-N/L, see Table 2). The DNA libraries of prokaryotic 16S rRNA genes and eukaryotic 18S rRNA genes were prepared by polymerase chain reaction (PCR) using 515F/805R [19] and 528F/706R primer pairs [17,18], respectively. An Illumina adapter was attached to the 5′ end of each primer. The PCR was performed with the following thermal cycling profile: an initial denaturation at 94 °C for 10 min, different numbers of amplification cycles (30 s each at 94 °C, 55 °C, and 72 °C) for prokaryotic (35–38 cycles) and eukaryotic (33 cycles) runs, and a final extension at 72 °C for 7 min. The resulting PCR products were sent to Macrogen Inc. (Seoul, Korea) for purification and sequencing on the Illumina MiSeq platform. Reads with low quality scores and ambiguous bases were discarded, and the filtered sequences were aligned and clustered into error-corrected amplicon sequence variants (ASVs) using DADA2 version 1.18.0 [20]. ASVs were rarefied by random sampling to the smallest library size to account for differences in sequencing depth. Taxonomic classification of ASVs was performed against the NCBI microbial database (≥85% query coverage or identity) using BLAST+ version 2.9.0 [21]. The sequence data generated in this study were deposited in the NCBI Sequence Read Archive (BioProject accession number PRJNA774114).

A quantitative matrix was generated based on the relative abundance of individual bacterial ASVs to the total bacterial reads in the prokaryotic 16S rRNA gene libraries. A cluster analysis was performed on the constructed matrix using the unweighted pair group method with the arithmetic means algorithm. Clustering calculations and dendrogram construction were carried out using the Bray-Curtis distance measure using PAST version 4.07 [22]. Nonmetric multidimensional scaling (NMS) ordination was performed on the matrix with the same distance measure using PC-ORD version 6 (MjM software, Gleneden Beach, OR, USA).

## 3. Results and Discussion

### 3.1. Culture pH Profiles

**Figure 1:**
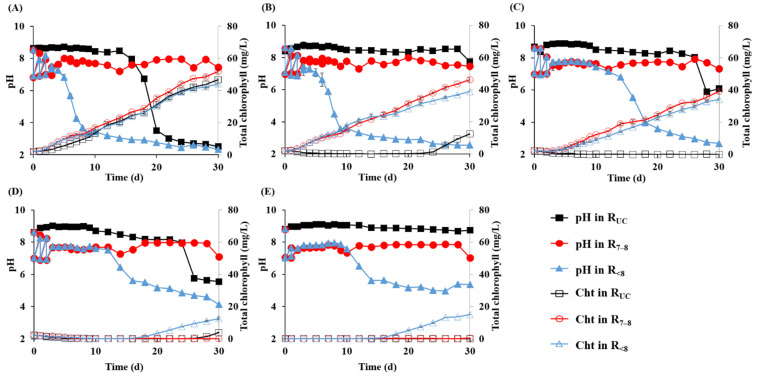
The profiles of pH and total chlorophyll concentration (Cht) during cultivation of the microalgae cultures with different pH control strategies (R_UC_, R_7__–8_, and R_<8_) at initial NH_4_^+^-N concentrations of 100, 200, 400, and 800 mg NH_4_^+^-N/L (**A**–**D**) and the uninoculated control cultures (**E**).

The pH of AD effluent is influenced by the substrate characteristics, operating conditions, digester performance, and other factors [23]. The initial pH of the AD effluent used in this study was 8.7, and those of the microalgae cultures ranged between 8.5 and 8.8 (Table 1). The pH tended to increase during the first 2 days in all of the cultures, and the R_7__–8_ and R_<8_ cultures were adjusted to the desired pH values by adding HCl solution (Figure 1). The R_UC_ cultures without pH control showed that the magnitude of pH increase tended to be greater at higher initial NH_4_^+^-N concentrations. Previous studies have explained a pH increase in microalgae cultures as a result of the volatilization of residual volatile fatty acids (VFAs) from the culture media [24] or the fixation of CO_2_ by microalgae [25]. However, these could not explain the pH increase because no residual VFAs were detected in all of the cultures (data not shown) and the uninoculated control cultures also showed an immediate pH increase. Given that the initial pH was slightly alkaline in all of the microalgae cultures, the loss of bicarbonate as CO_2_ under continuous aeration appears to have led to the pH increase during the early period of cultivation [26]. In support of this, the experimental cultures contained a good amount of inorganic carbon (73.5–462.9 mg/L), in inverse proportion to the dilution rate of the AD effluent (Table 1).

The pH remained above 8.5 in all of the R_UC_ cultures until Day 14, but thereafter, the pH of the R_UC_-100 cultures (i.e., the R_UC_ cultures at an initial NH_4_^+^-N concentration of 100 mg/L) dropped suddenly below 3 in about a week (Figure 1). Although in less magnitude, the other R_UC_ cultures also showed a significant decrease in pH after Day 25, except the uninoculated control (R_UC_-C). The decrease in culture pH during cultivation was more pronounced in the R_<8_ cultures. All of the R_<8_ cultures, including R_<8_-C, showed a more rapid and significant decrease in pH than the R_UC_ cultures at the same initial NH_4_^+^-N concentrations. The culture pH declined below 3 during cultivation for 30 days in the R_<8_ cultures at 400 mg NH_4_^+^-N/L or less, and especially R_<8_-100 and R_<8_-200 reached pH 3 by Day 10. The R_7__–8_ cultures also tended to acidify, but the pH was maintained neutral by adding NaOH solution. The decreasing pH of R_UC_ and R_<8_ cultures can be attributed to the consumption of alkalinity due to the ammonium uptake and proton release by microalgae during their growth [11]. NH_4_^+^-N was the dominant form of available nitrogen for microalgae in the AD effluent, without detectable amounts of nitrite or nitrate, the utilization of which by microalgae produces alkalinity. Accordingly, among the R_UC_ or R_<8_ cultures, the cultures with greater pH decreases showed higher microalgal growth (Figure 1).

### 3.2. Microalgal Growth and Nutrient Removal

**Figure 2:**
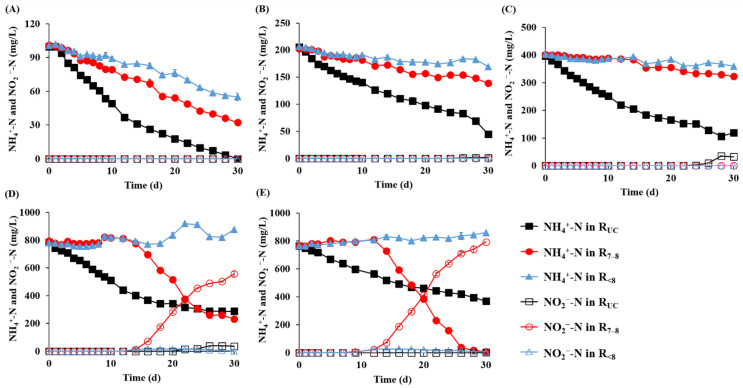
The NH_4_^+^-N and NO_2_^−^-N concentration profiles during cultivation of the microalgae cultures with different pH control strategies (R_UC_, R_7__–8_, and R_<8_) at initial NH_4_^+^-N concentrations of 100, 200, 400, and 800 mg NH_4_^+^-N/L (**A**–**D**) and the uninoculated control cultures (**E**).

The chlorophyll concentration increased continuously and significantly during cultivation in the R_7__–8_ and R_<8_ cultures at 400 mg NH_4_^+^-N/L or less and R_UC_-100 (>30 mg/L on Day 30), while all the other cultures showed limited or no increase (Figure 1). The optical density profiles were very similar to the chlorophyll concentration profiles (Figure S1). These results indicate that the growth of microalgae was inhibited under high NH_4_^+^-N conditions [27]. Accordingly, the increase in chlorophyll concentration was greater in the cultures at lower initial NH_4_^+^-N concentrations regardless of pH control strategy. Little increase in chlorophyll in R_UC_-200 and R_UC_-400 appears to reflect the inhibition of microalgal growth due to the evolution of toxic NH_3_ (pKa = 9.25) under alkaline conditions [28], given that their pH remained alkaline between 8.3–9.0 for more than 24 days from the start of cultivation (Figure 1). Another possibility to consider is that the loss of volatile free ammonia during aerated cultivation could limit the amount of nitrogen available to microalgae. The volatilization loss of NH_3_ from the R_UC_ cultures was evident and substantial. All of the R_UC_ cultures—even those with little or no increase in chlorophyll concentration—showed an immediate and fast decrease in NH_4_^+^-N concentration, and the highest nitrogen removal rate and efficiency were observed in the R_UC_ cultures at all of the initial NH_4_^+^-N concentrations (Figure 2). These results indicate that a large proportion of nitrogen was removed abiotically in the R_UC_ cultures. Accordingly, the contribution of NH_3_ volatilization to total nitrogen removal calculated from the decrease in total nitrogen concentration during cultivation ranged from 29.6 to 71.0% in the R_UC_ cultures, while it was below 5% in the R_7__–8_ and R_<8_ cultures (Table 2). The significantly lower NH_3_ volatilization rate in R_UC_-100 than in the other R_UC_ cultures can be attributed to the drastic decrease in pH after Day 14 with the growth of microalgae (Figure 1). Nitrogen removal through NH_3_ volatilization is not desirable in microalgae cultivation because it reduces nutrient availability and limits microalgal growth. The concentration profiles of PO_4_^3−^-P, another important nutrient for the growth of microalgae, also indicated a significant NH_3_ volatilization in the R_UC_ cultures. PO_4_^3−^-P was completely removed only in the cultures where a significant increase in chlorophyll concentration was observed (Figure S2), evidencing that the NH_4_^+^-N removal in R_UC_ cultures (especially those at initial NH_4_^+^-N concentrations of ≥200 mg/L) was primarily due to NH_3_ volatilization rather than microalgal growth.

**Figure 3:**
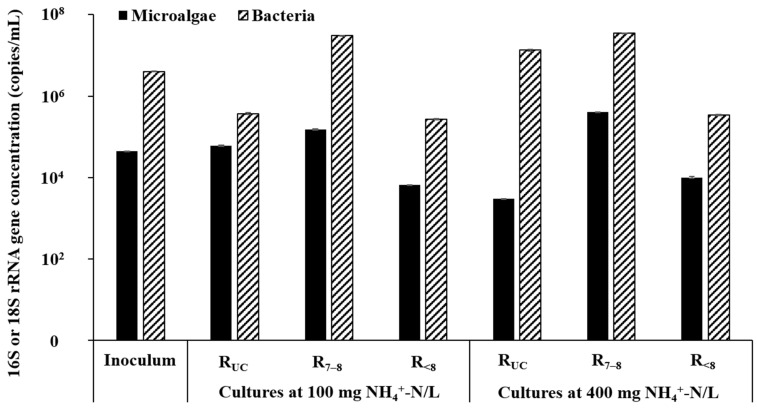
The concentrations of microalgal 18S rRNA genes and bacterial 16S rRNA genes in the inoculum and selected microalgae cultures (on Day 30).

In the initial NH_4_^+^-N concentration range of 100–400 mg/L, the chlorophyll concentration increased the most in the R_7__–8_ cultures (Figure 1), and accordingly, the NH_4_^+^-N removal was greater in the R_7__–8_ cultures than in the R_<8_ cultures (Figure 2). This result shows that a neutral pH is optimal for the growth and nitrogen uptake of the inoculated microalgae [29]. Accordingly, the biological NH_4_^+^-N uptake during cultivation, which was calculated by subtracting the volatilization loss of NH_3_-N and the production of NO_2_^−^-N and NO_3_^−^-N by ammonia oxidation from the measured NH_4_^+^-N removal, was significantly greater in the R_7__–8_ cultures than in the R_<8_ cultures (Table 2). Of note is that NO_2_^−^-N accumulated to a significant level with a concomitant decrease in NH_4_^+^-N during the latter half of cultivation in the R_7__–8_-800 and R_7__–8_-C cultures but not in the other cultures (Figure 2). In these R_7__–8_ cultures, the removal of NH_4_^+^-N corresponded stoichiometrically to the formation of NO_2_^−^-N, indicating that NH_4_^+^ was removed by bacterial oxidation to NO_2_^−^ with little contribution of microalgae. This observation implies that ammonia-oxidizing bacteria (AOBs), possibly derived from both the inoculum and the AD effluent, outcompeted microalgae for NH_4_^+^-N under neutral nutrient-rich conditions [30]. The R_7__–8_-800 and R_7__–8_-C cultures were cultivated with the least diluted AD effluent, and therefore, most likely had a higher initial population of AOBs than the other R_7__–8_ cultures. This may also help to explain the significant accumulation of NO_2_^−^-N in these cultures only. Given that uninoculated R_7__–8_-C showed a significant NO_2_^−^-N buildup, AOBs were likely derived primarily from the AD effluent. In addition, the growth of microalgae was likely further inhibited by the accumulation of toxic NO_2_^−^ formed by AOBs [31]. In contrast to the observation in R_7__–8_-800 and R_7__–8_-C cultures, the chlorophyll concentration increased without nitrite accumulation after Day 16 in the R_<8_-800 and R_<8_-C cultures (Figure 2 and Figure 3). This result appears to reflect the adaptation and growth of microalgae under acidic conditions which inhibited the growth of AOBs in the cultures. Particularly, the microalgal growth in the R_<8_-C culture was most likely due to the enrichment of indigenous microalgae in the AD effluent [5].

**Figure 4:**
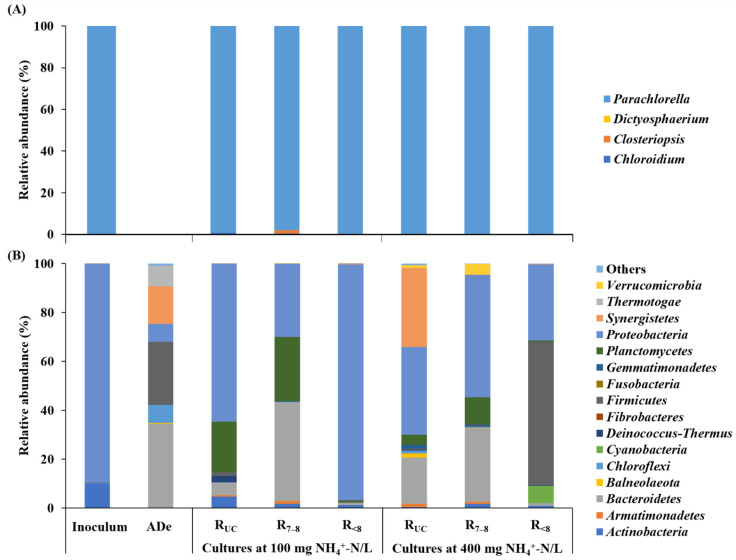
Taxonomic distribution of microalgal sequences at the genus level (**A**) and bacterial sequences at the phylum level (**B**) in the rRNA gene libraries of the inoculum and selected microalgae cultures (on Day 30). Sequences with relative abundance less than 1% were classified as “Others”. ADe, raw anaerobic digestion effluent.

For further insight into the growth of microalgae and bacteria in the cultures under different conditions, the concentrations of microalgal 18S rRNA genes and bacterial 16S rRNA genes at the end of cultivation were determined for the cultures at initial NH_4_^+^-N concentrations of 100 and 400 mg/L. Due to the lack of primers to selectively detect microalgae, the concentration of microalgal 18S rRNA genes was estimated by multiplying the concentration of eukaryotic 18S rRNA genes measured by dPCR by the fraction of microalgal sequences in a eukaryotic library analyzed by HTS using the same eukaryote-specific primer pair (see Section 2.3 and Section 2.4 for details). Corresponding to the chlorophyll concentration profiles, the highest abundance of microalgae was observed in the R_7__–8_ cultures at both initial NH_4_^+^-N concentrations, while the R_UC_-400 culture without apparent microalgal growth showed the lowest abundance of microalgae among the six cultures analyzed (Figure 2 and Figure 4). Bacterial abundance was also markedly higher in the R_7__–8_ cultures than in the other cultures, confirming that neutral conditions were optimal for the growth of both microalgae and bacteria. Meanwhile, the R_<8_ cultures showed the lowest bacterial abundance, likely due to the inhibition of bacterial growth under acidic conditions. The microalgae-to-bacteria rRNA gene ratio was three to four-fold higher in the R_<8_ cultures than in the R_7__–8_ cultures, suggesting that the pH control strategy used in the R_<8_ cultures could limit bacterial consumption of nutrients and could provide an environment allowing microalgae to better compete with bacteria including AOBs.

### 3.3. Biomass Production and Harvesting

A promising use of microalgal biomass cultivated in wastewater is as a feedstock for the production of renewable fuels and chemicals [7,8]. Therefore, efficient biomass production and harvesting can improve the economic feasibility of microalgal treatment of AD effluents. The biomass yields, calculated from the increased concentrations of volatile suspended solids (Y_VSS_) and chlorophyll (Y_Cht_) per unit of NH_4_^+^-N supplied, were both highest in the R_7__–8_ cultures at all of the initial NH_4_^+^-N concentrations where visible microalgal growth was observed during cultivation (Table 3). Meanwhile, the Y_Cht_/Y_VSS_ ratio was significantly higher in the R_<8_ cultures than in the other cultures. These results indicated that the growth of microalgae (and also bacteria) was most active under neutral conditions, while the relative dominance of microalgae (over bacteria) in culture microbial community was greater under acidic conditions. This observation reflects the more pronounced inhibition of the growth of bacteria than that of microalgae by acidic pH, which agrees well with the dPCR quantification results of microalgal and bacterial abundances (Figure 3). It appears that maintaining neutral culture pH is suitable for high biomass production, while acidic pH control is advantageous for more selective growth of microalgal biomass. Note that the chemical consumption for pH adjustment, which should be much higher for the R_7__–8_ cultures than for the R_<8_ cultures, needs to be considered when applying the pH control strategies.

Biomass harvesting accounts for at least 20–30% of the total cost for producing microalgal biomass [32]. The different pH control strategies also affect the settleability of cultivated biomass, which is an important factor determining the harvesting efficiency (Table S1). Biomass settleability was determined for the cultures which showed visible microalgal growth. The 24-h settleability ranged from 59.7 to 97.3% among the cultures, while being markedly higher in the R_<8_ cultures (≥93.3%) than in the other cultures (≤81.8%) at all initial NH_4_^+^-N concentrations. The difference was even more pronounced for 12-h settleability, especially at lower NH_4_^+^-N loads. These results indicated that culture pH had a significant effect on the settleability of cultivated biomass, and previous studies have reported that biomass settling was improved under acidic or alkaline conditions by auto- or bioflocculation [32,33]. Meanwhile, R_UC_-100 with similar end pH but shorter exposure to acidic pH showed significantly lower biomass settleability (81.8% after 24 h) than the R_<8_ cultures, although it was highest among the R_UC_ and R_7__–8_ cultures. Therefore, acidic end pH could not solely explain the higher settleability of R_<8_ biomass, and the inhibition of bacterial growth and resulting changes in microbial community composition during cultivation under acidic conditions may also have contributed.

### 3.4. Microbial Community Structure

HTS analysis was performed on the inoculum, AD effluent, and cultures at initial NH_4_^+^-N concentrations of 100 and 400 mg/L, and a total of 1,386,408 eukaryotic 18S rRNA gene reads (154,045 ± 14,997 reads/sample) and 2,023,130 prokaryotic 16S rRNA gene reads (203,572 ± 23,812 reads/sample) were obtained. Microalgal reads were abundant in all of the eukaryotic libraries analyzed except that of AD effluent where none was found. All of the retrieved microalgal sequences were classified to the phylum *Chlorophyta* (green algae), and almost all of them were clustered into one ASV assigned to the genus *Parachlorella* (98.0–100% in the inoculum and all of the culture samples analyzed, Figure 4A). Microalgae belonging to the family *Chlorellaceae*, for example, *Chlorella* and *Parachlorella* species, are widely distributed in the environment and have been cultivated in different wastewaters [34,35]. *Parachlorella* species are characterized by their high tolerance to environmental stresses, such as oxidative stress, high salt concentrations, and temperature and pH changes [36,37,38], enabling them to grow well in wastewater environments. These results suggested that the *Parachlorella* population derived from the inoculum effectively adapted and proliferated in the cultures, even under highly acidic conditions (Figure 1). In support of this, members of the genus have been isolated from an extremely acidic geothermal pond of pH 2.5–2.8 [39] and an acidic peat bog lake of pH 3.95 [40]. In addition, Shimura, et al. [41] reported that a *Parachlorella* strain isolated from activated sludge from a wastewater treatment plant (approximately 78 km from the Fukushima Daiichi Nuclear Power Plant) was viable in a wide range of pH (3–11) and temperatures (up to 60 °C) and able to grow in both fresh and sea water. Accordingly, the facts that active microalgal growth was observed in the R_7__–8_ cultures as well as the R_<8_ cultures and that the inoculum originated from the sludge of a digester run at circumneutral pH, indicate that the cultivated *Parachlorella* has a wide pH range for growth.

Prokaryotic sequences were clustered into 979 bacterial and 13 archaeal ASVs. Archaeal reads accounted for less than 0.1% of prokaryotic reads in all of the cultures examined, and they were excluded from further analysis. The taxonomic affiliation of major bacterial ASVs (≥3% of the total bacterial reads in at least one library) is presented in Table 4. Twenty-three bacterial phyla were identified in total, and *Bacteroidetes*, *Firmicutes*, *Planctomycetes*, *Proteobacteria*, and *Synergistetes* were the major ones (Figure 4B). However, their relative dominance varied greatly among the samples analyzed. The inoculum bacterial community consisted almost entirely of *Proteobacteria* (89.2%) and *Actinobacteria* (10.2%), while the AD effluent had a significantly more diverse and even bacterial community, with *Bacteroidetes*, *Firmicutes*, and *Synergistetes* being the most abundant phyla. This result seems to reflect the differences in culture conditions between their sources: aerated microalgae enrichment for the former and mixed-culture AD for the latter. *Dyella* (ASV B32) and *Paraburkholderia* (ASV B27) genera belonging to the phylum *Proteobacteria* together accounted for 83.1% of the total bacterial sequences in the inoculum library. Members of these genera often coexist with microalgae in mixed-culture environments and have been suggested to promote the growth of microalgae [5,42]. *Proteobacteria* (ASVs B17–B34) occurred as a major phylum in all of the cultures analyzed, and particularly, the families *Burkholderiaceae*, *Rhodobacteraceae*, and *Xanthomonadaceae*, whose members are commonly associated with microalgae [43,44,45], were identified in abundance across the cultures. In contrast, *Bacteroidetes* (ASVs B1–B8), whose members are also more likely to interact with green algae than other bacteria [46], showed very low relative abundance in the R_<8_-100 and R_<8_-400 cultures (≤0.1%) as compared with those in the other cultures (2.1–34.2%), especially those maintained under neutral conditions (≥18.1%). This result, together with the culture pH profiles (Figure 1), suggests that the long-term exposure to highly acidic pH (<3) inhibited the growth of *Bacteroidetes* [47]. Similarly, *Planctomycetes* (ASVs from B14 to B16) occurred in much higher relative abundance in the R_UC_ and R_7__–8_ cultures than in the R_<8_ cultures. Members of this phylum, especially of the order *Pirellulales* (ASV B16), are common in both microalgae- and macroalgae-associated bacterial communities [48,49,50]. Meanwhile, phototrophic *Cyanobacteria*, which coexist and interplay with microalgae [46], was found in notable relative abundance (7.0%) only in R_<8_-400.

**Figure 5:**
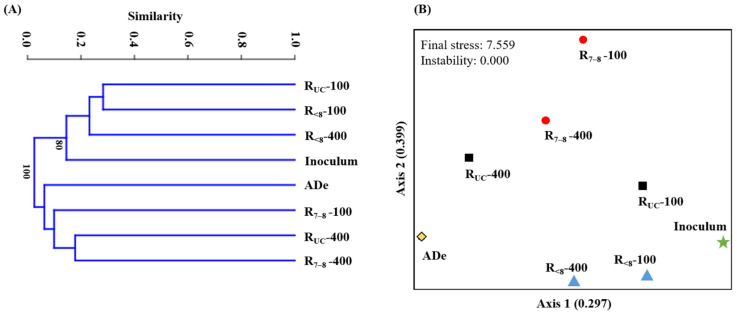
Cluster dendrogram (**A**) and nonmetric multidimensional scaling plot (**B**) constructed based on the distribution of bacterial amplicon sequence variants. Bootstrap values of 70% or higher (1000 replicates) are shown at the nodes. Analyzed bacterial communities are labeled with the corresponding culture names. ADe, raw anaerobic digestion effluent.

These results suggest that bacteria derived from the inoculum and AD effluent likely affected the growth of microalgae and contributed to the removal of nutrients, although difficult to determine the extent of their influence, under different culture conditions. Accordingly, the analyzed bacterial community structures were roughly separated according to both the strength of AD effluent (along Axis 1) and the culture pH (along Axis 2) in the NMS plot, while sharing limited similarities among them (Figure 5). As expected from the reaction profiles, the bacterial community profiles of the R_7__–8_ and R_<8_ cultures were most distantly related from each other. At both initial NH_4_^+^-N concentrations of 100 and 400 mg/L, species richness (i.e., the number of ASVs), the Shannon index, and the inverse Simpson index were significantly lower in the R_<8_ cultures than in the other cultures (Table S2). This finding indicates that the long-term exposure to highly acidic conditions affected not only the abundance but also the diversity of bacteria in the experimental cultures.

## 4. Conclusions

The inoculum mixed-culture microalgae dominated by *Parachlorella* effectively removed NH_4_^+^-N and grew well in AD effluent, even at pH below 3 caused by the acidification of culture with microalgal growth. A significant volatilization loss of NH_3_ occurred during cultivation in uncontrolled pH cultures (R_UC_). Biological NH_4_^+^-N removal and microalgal growth were highest in the cultures maintained at neutral pH (R_7__–8_), while more selective growth of microalgae over bacteria and better biomass settleability were achieved in the cultures controlled below pH 8 (R_<8_). A neutral pH control seemed to be suitable for high biomass production, while controlling the culture pH below 8 led to a preferential growth of microalgae (i.e., low bacterial co-existence or contamination). Further, the pH control strategies influenced the development of microbial communities. The results suggest that more attention needs to be paid to culture pH and that its proper control in microalgae is advantageous for more selective growth of microalgal biomass cultures using wastewater, especially at the field scale, and for optimum growth of microalgae with minimum loss of NH_3_.

## Figures and Tables

**Table 1:** Physicochemical characteristics of microalgae inoculum, raw AD effluent, and experimental cultures at time 0.

Culture or Sample	Total Carbon (mg/L)	Inorganic Carbon (mg/L)	Total Nitrogen (mg/L)	NH_4_^+^-N (mg/L)	pH
Microalgae inoculum	44.2 ± 0.8	0.0 ± 0.0	16.8 ± 0.3	12.7 ± 0.0	2.8 ± 0.0
Raw AD effluent	1285.1 ± 23.9	1066.3 ± 8.1	1841.7 ± 31.0	1848.2 ± 0.0	8.7 ± 0.0
100-mg NH_4_^+^-N/L test cultures	103.8 ± 2.5	73.5 ± 0.5	100.0 ± 0.2	100.3 ± 0.6	8.6 ± 0.1
200-mg NH_4_^+^-N/L test cultures	189.5 ± 5.7	154.7 ± 1.7	202.1 ± 1.4	205.0 ± 2.3	8.5 ± 0.1
400-mg NH_4_^+^-N/L test cultures	356.7 ± 9.4	267.8 ± 1.9	422.7 ± 3.2	400.6 ± 3.5	8.6 ± 0.0
800-mg NH_4_^+^-N/L test cultures	613.7 ± 25.7	462.9 ± 1.0	817.0 ± 1.3	786.0 ± 6.8	8.6 ± 0.0
Uninoculated control cultures ^a^	568.2 ± 17.4	442.4 ± 20.4	810.3 ± 3.2	765.1 ± 0.0	8.8 ± 0.0

^a^ Cultivated at an initial NH_4_^+^-N concentration of 800 mg/L.

**Table 2:** Removal of total and ammonia nitrogen during cultivation.

Culture	TN_0_ (mg/L)	TN_30_ (mg/L)	AV ^a^ (%)	AR ^b^ (mg/L)	AO ^c^ (mg/L)	BAU ^d^ (mg/L)
R_UC_-100	99.8 ± 4.2	70.3 ± 1.0	29.6	99.7 ± 0.0	– ^e^	70.2 ± 4.4
R_7__–8_-100	100.1 ± 1.0	100.6 ± 0.2	–	68.6 ± 0.2	–	68.6 ± 0.2
R_<8_-100	100.2 ± 1.4	109.2 ± 0.8	–	45.5 ± 3.2	–	45.5 ± 3.2
R_UC_-200	202.0 ± 2.4	58.6 ± 4.8	71.0	160.8 ± 0.2	1.4 ± 0.0	16.0 ± 5.3
R_7__–8_-200	200.8 ± 3.4	191.4 ± 2.9	4.7	63.9 ± 1.0	–	63.9 ± 1.0
R_<8_-200	203.6 ± 2.2	193.8 ± 8.7	4.8	37.6 ± 2.1	–	37.6 ± 2.1
R_UC_-400	419.2 ± 5.7	160.1 ± 5.0	61.8	278.1 ± 1.0	33.0 ± 1.0	–
R_7__–8_-400	425.4 ± 3.8	414.5 ± 0.4	2.6	79.4 ± 0.5	–	68.5 ± 3.9
R_<8_-400	423.6 ± 0.1	416.9 ± 5.4	1.6	43.4 ± 1.5	–	43.4 ± 1.5
R_UC_-800	815.5 ± 6.2	353.4 ± 1.8	56.7	496.1 ± 0.9	37.9 ± 0.2	–
R_7__–8_-800	817.8 ± 5.7	791.5 ± 2.4	3.2	563.3 ± 1.9	567.1 ± 1.8	–
R_<8_-800	817.8 ± 1.6	802.4 ± 0.5	1.9	–	3.0 ± 0.2	–
R_UC_-C	807.1 ± 0.2	348.6 ± 1.7	56.8	396.7 ± 0.6	1.9 ± 0.0	–
R_7__–8_-C	813.4 ± 4.0	779.8 ± 1.2	4.1	757.9 ± 0.2	801.3 ± 0.6	–
R_<8_-C	810.5 ± 4.6	862.7 ± 3.2	–	–	7.1 ± 0.1	–

TN_0_, the initial total nitrogen (TN) concentration at time 0; TN_30_, the residual TN concentration after 30-day cultivation; AV, ammonia volatilization; AR, ammonium removal; AO, ammonia oxidation; BAU, biological ammonium uptake. ^a^ (TN_0_ − TN_30_)/TN_0_ × 100. ^b^ The decrease in NH_4_^+^-N concentration during cultivation for 30 days. ^c^ The increase in the sum of NO_2_^–^-N and NO_3_^–^-N concentrations during cultivation for 30 days. ^d^ AR − AV − AO. ^e^ Zero or below.

**Table 3:** Production and yield of biomass and chlorophyll during the cultivation.

	Biomass Production		Biomass Yield		Preferentiality
	P_VSS_ (mg/L) ^a^	P_Cht_ (mg/L) ^b^	Y_VSS_ (g/g) ^c^	Y_Cht_ (g/g) ^d^	Y_Cht_/Y_VSS_
R_UC_-100	1050.0 ± 0.0	44.8 ± 0.8	10.53 ± 0.00	0.45 ± 0.00	0.043 ± 0.000
R_7__–8_-100	1180.0 ± 28.3	49.9 ± 1.1	11.71 ± 0.01	0.50 ± 0.00	0.042 ± 0.000
R_<8_-100	895.0 ± 21.2	42.0 ± 1.3	8.90 ± 0.00	0.42 ± 0.00	0.047 ± 0.000
R_UC_-200	340.0 ± 0.0	10.4 ± 0.2	1.65 ± 0.00	0.05 ± 0.00	0.031 ± 0.000
R_7__–8_-200	1025.0 ± 25.5	44.1 ± 1.2	5.06 ± 0.00	0.22 ± 0.00	0.043 ± 0.000
R_<8_-200	785.0 ± 35.4	36.6 ± 0.1	3.79 ± 0.01	0.18 ± 0.00	0.047 ± 0.000
R_UC_-400	– ^e^	–	–	–	–
R_7__–8_-400	720.0 ± 28.3	36.9 ± 1.2	1.79 ± 0.00	0.09 ± 0.00	0.051 ± 0.000
R_<8_-400	525.0 ± 15.8	32.1 ± 0.6	1.30 ± 0.00	0.08 ± 0.00	0.061 ± 0.000
R_UC_-800	–	1.7 ± 0.3	–	0.00 ± 0.00	–
R_7__–8_-800	–	–	–	–	–
R_<8_-800	–	10.2 ± 0.7	–	0.01 ± 0.00	–
R_UC_-C	–	–	–	–	–
R_7__–8_-C	–	–	–	–	–
R_<8_-C	–	15.1 ± 0.2	–	0.02 ± 0.00	–

^a^, The increase in volatile suspended solids (VSS) concentration during the cultivation for 30 days; ^b^, the increase in total chlorophyll (Cht) concentration during the cultivation for 30 days; ^c^, the amount of VSS produced per unit mass of initial NH_4_^+^-N (= P_VSS_/Initial NH_4_^+^-N concentration); ^d^, the amount of Cht produced per unit mass of initial NH_4_^+^-N (= P_Cht_/Initial NH_4_^+^-N concentration); ^e^, zero or below.

**Table 4:** Relative abundance and taxonomic affiliation of major bacterial ASVs (≥3% relative abundance in at least one library).

ASV	Taxonomy ^a^	Ino	ADe	R_UC_- 100	R_7__–8_- 100	R_<8_- 100	R_UC_- 400	R_7__–8_- 400	R_<8_- 400	Closest Species (Nucleotide Accession Number) ^b^	Sim (%)
B1	*Longimonas*	– ^c^	–	0.7	3.3	–	1.5	2.5	–	*Longimonas halophila* (NR136497)	86.6
B2	*Dysgonomonas*	0.0	7.9	–	–	–	0.0	–	0.0	*Dysgonomonas capnocytophagoides* (NR113133)	91.7
B3	*Alistipes*	0.0	17.7	–	–	–	0.0	0.0	–	*Alistipes communis* (NR133025)	85.9
B4	*Edaphocola*	0.0	0.7	–	0.4	–	0.0	3.9	–	*Edaphocola flava* (NR165730)	93.7
B5	*Terrimonas*	–	–	0.1	4.7	0.1	–	0.2	–	*Terrimonas rubra* (NR109417)	95.3
B6	*Emticicia*	–	–	–	23.9	0.0	0.0	–	–	*Emticicia soli* (NR157720)	100.0
B7	*Rhabdobacter*	–	–	0.5	1.9	–	1.2	5.6	0.0	*Rhabdobacter roseus* (NR147750)	93.4
B8	*Aequorivita*	0.0	0.0	0.8	–	–	0.1	5.9	0.0	*Aequorivita viscosa* (NR109011)	99.6
B9	*Leptolinea*	0.0	4.9	–	–	0.0	0.0	0.0	0.1	*Leptolinea tardivitalis* (NR040971)	91.3
B10	*Limnoraphis*	–	–	0.1	–	0.2	–	–	7.0	*Limnoraphis robusta* (NR118325)	87.1
B11	*Alicyclobacillus*	–	–	–	–	–	–	–	57.5	*Alicyclobacillus acidiphilus* (NR028637)	98.0
B12	*Ercella*	0.0	3.4	0.0	–	–	–	–	0.6	*Ercella succinigenes* (NR134026)	86.6
B13	*Syntrophomonas*	–	3.9	0.0	–	–	–	–	–	*Syntrophomonas bryantii* (NR104881)	97.2
B14	*Fimbriiglobus*	–	–	0.0	14.9	0.0	–	0.1	–	*Fimbriiglobus ruber* (NR148575)	95.7
B15	*Gemmata*	0.0	–	–	7.8	–	0.1	–	–	*Gemmata massiliana* (NR148576)	95.7
B16	*Bremerella*	–	–	20.3	0.0	–	2.5	5.4	0.2	*Bremerella volcania* (NR169319)	92.5
B17	*Brevundimonas*	–	0.0	1.3	0.1	–	1.2	3.3	–	*Brevundimonas subvibrioides* (NR112028)	100.0
B18	*Caulobacter*	–	–	0.1	1.9	–	0.0	4.9	–	*Caulobacter daechungensis* (NR118485)	100.0
B19	*Phenylobacterium*	0.2	–	1.5	0.0	6.4	0.0	0.0	0.9	*Phenylobacterium zucineum* (NR074119)	96.4
B20	*Bradyrhizobium*	0.2	–	1.2	0.0	6.8	0.0	0.1	2.1	*Bradyrhizobium ganzhouense* (NR133706)	100.0
B21	*Variibacter*	0.9	–	4.6	0.2	21	0.1	0.1	8.7	*Variibacter gotjawalensis* (NR134225)	96.0
B22	*Devosia*	–	–	0.0	7.2	–	–	0.8	–	*Devosia humi* (NR147759)	100.0
B23	*Oceaniglobus*	–	–	–	0.1	–	0.8	6.4	–	*Oceaniglobus indicus* (NR159234)	100.0
B24	*Reyranella*	–	–	15.5	5.4	–	0.0	0.5	–	*Reyranella aquatilis* (NR158037)	100.0
B25	*Acidisoma*	1.0	–	–	–	3.4	–	–	–	*Acidisoma tundrae* (NR042705)	98.8
B26	*Cupriavidus*	0.3	–	2.0	0.0	12.8	0.0	–	2.1	*Cupriavidus metallidurans* (NR074704)	100.0
B27	*Paraburkholderia*	37.7	–	–	–	1.2	–	–	–	*Paraburkholderia xenovorans* (NR118083)	100.0
B28	*Ralstonia*	0.4	–	2.6	0.1	10.5	0.0	–	5.6	*Ralstonia pickettii* (NR113352)	100.0
B29	*Janthinobacterium*	–	–	0.7	–	3.6	–	–	0.4	*Janthinobacterium lividum* (NR164625)	100.0
B30	*Sandaracinus*	–	–	–	0.8	0.0	0.4	3.0	–	*Sandaracinus amylolyticus* (NR118001)	99.2
B31	*Pseudomonas*	0.1	–	1.1	0.0	3.4	–	–	0.6	*Pseudomonas veronii* (NR112075)	100.0
B32	*Dyella*	45.4	–	17.5	–	6.9	–	–	2.1	*Dyella flava* (NR157998)	99.2
B33	*Luteimonas*	–	–	–	–	0.1	–	10.9	0.1	*Luteimonas abyssi* (NR133773)	100.0
B34	*Pseudoxanthomonas*	–	–	–	–	–	16.7	1.2	0.0	*Pseudoxanthomonas daejeonensis* (NR113984)	95.7
B35	*Thermovirga*	0.1	9.4	0.1	–	0.3	29.5	0.1	–	*Thermovirga lienii* (NR043522)	93.7
B36	*Mesotoga*	–	4.5	–	–	–	–	–	–	*Mesotoga infera* (NR118572)	100.0
B37	*Mesotoga*	0.1	3.9	–	–	–	0.0	0.0	–	*Mesotoga prima* (NR102952)	100.0

Cells with relative abundance values are colored in a heatmap-like fashion. ASV, amplicon sequence variant; Ino, microalgae inoculum; ADe, raw anaerobic digestion effluent; Sim, sequence similarity (85% cutoff); ^a^, taxonomic assignment at the genus level by BLAST+ against the National Center for Biotechnology Information (NCBI) database; ^b^, closest species identified by BLAST+ against the NCBI database; ^c^, not detected at all (a zero read). Note that ”0.0” means a non-zero read but in very low relative abundance (<0.1%).

## Data Availability

The data presented in this study are available in article.

## Supplementary Materials

The following supporting information can be downloaded at: https://www.mdpi.com/article/10.3390/microorganisms10020357/s1, Figure S1: The optical density profiles during cultivation of the microalgae cultures with different pH control strategies (R_UC_, R_7–8_, and R_<8_) at initial NH_4_^+^-N concentrations of 100, 200, 400, and 800 mg NH_4_^+^-N/L (A–D) and the uninoculated control cultures (E), Figure S2: The PO_4_^3-^-P concentration profiles during cultivation of the microalgae cultures with different pH control strategies (R_UC_, R_7–8_, and R_<8_) at initial NH_4_^+^-N concentrations of 100, 200, 400, and 800 mg NH_4_^+^-N/L (A–D) and the uninoculated control cultures (E), Table S1: Settleability of cultivated biomass, Table S2: Richness and diversity indices of bacterial communities based on the ASV profiles.
