# Supplementary material for: Effects of Different pH Control Strategies on Microalgae Cultivation and Nutrient Removal from Anaerobic Digestion Effluent
Source: Microorganisms. 2022 Feb 3;10(2):357. doi: 10.3390/microorganisms10020357 (PMC8879683; doi:10.3390/microorganisms10020357)
Supplement: Supplementary file 1 [file microorganisms-10-00357-s001.zip › microorganisms-1535286-supplementary.pdf]

Effects of different pH control strategies on microalgae cultivation and nutrient removal from anaerobic digestion effluent

Hyeonjung Yu[1], Jaai Kim[1], Chaeyoung Rhee[2], Juhee Shin[2], Seung Gu Shin[2], Changsoo Lee[1,*]

[1]Department of Urban and Environmental Engineering, Ulsan National Institute of Science and Technology (UNIST), 50 UNIST-gil, Eonyang-eup, Ulju-gun, Ulsan 44919, Republic of Korea

[2]Department of Energy Engineering, Future Convergence Technology Research Institute, Gyeongsang National University, 501 Jinju-daero, Jinju, Gyeongnam 52828, Republic of Korea

* Corresponding author.

Tel.: +82 52 217 2822; Fax: +82 52 217 2819; E-mail address: cslee@unist.ac.kr.

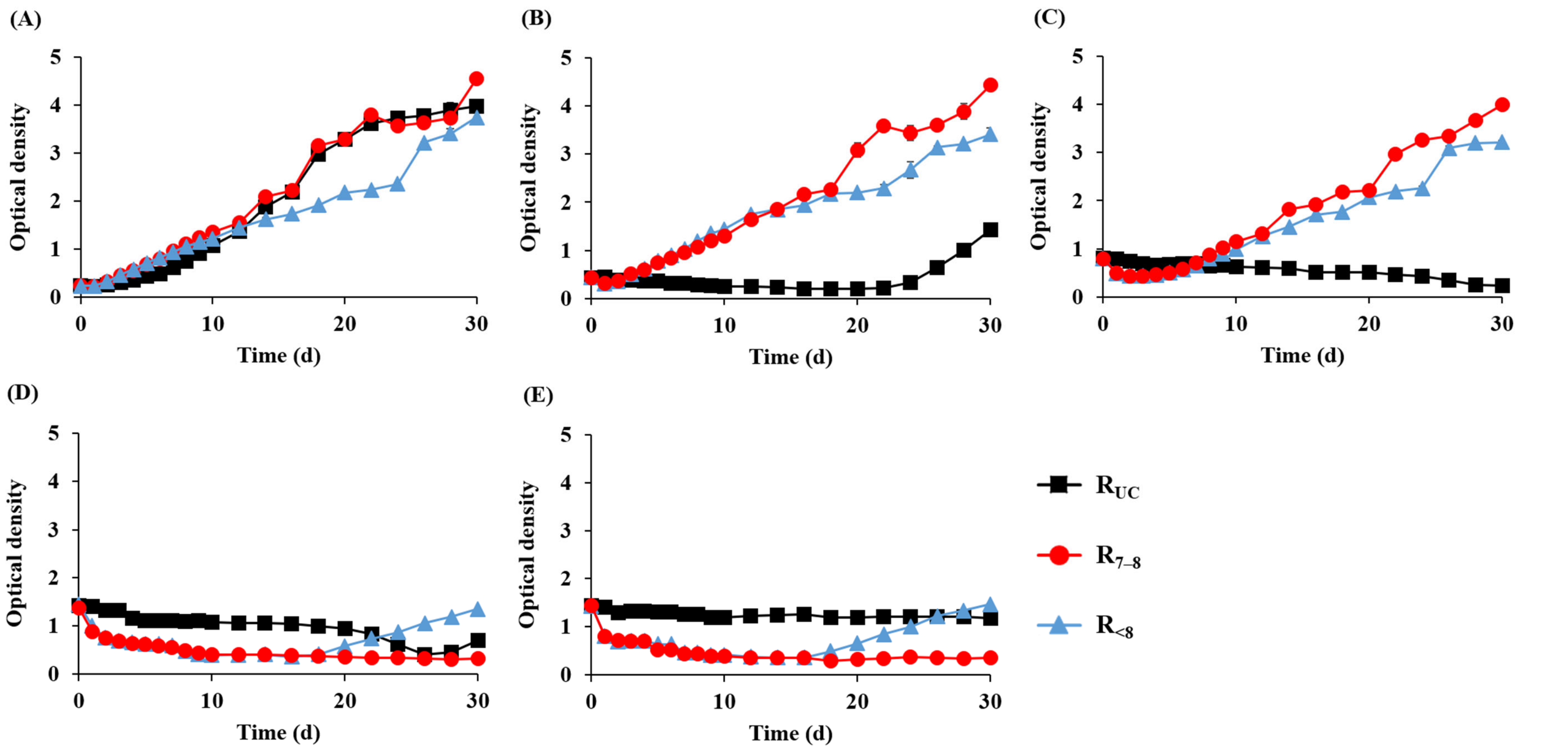

Figure S1. The optical density profiles during cultivation of the microalgae cultures with different pH control strategies (R_{UC}, $R_{7–8}$, and $R_{<8}$) at initial NH_4^+-N concentrations of 100, 200, 400, and 800 mg NH_4^+-N/L (A–D) and the uninoculated control cultures (E).

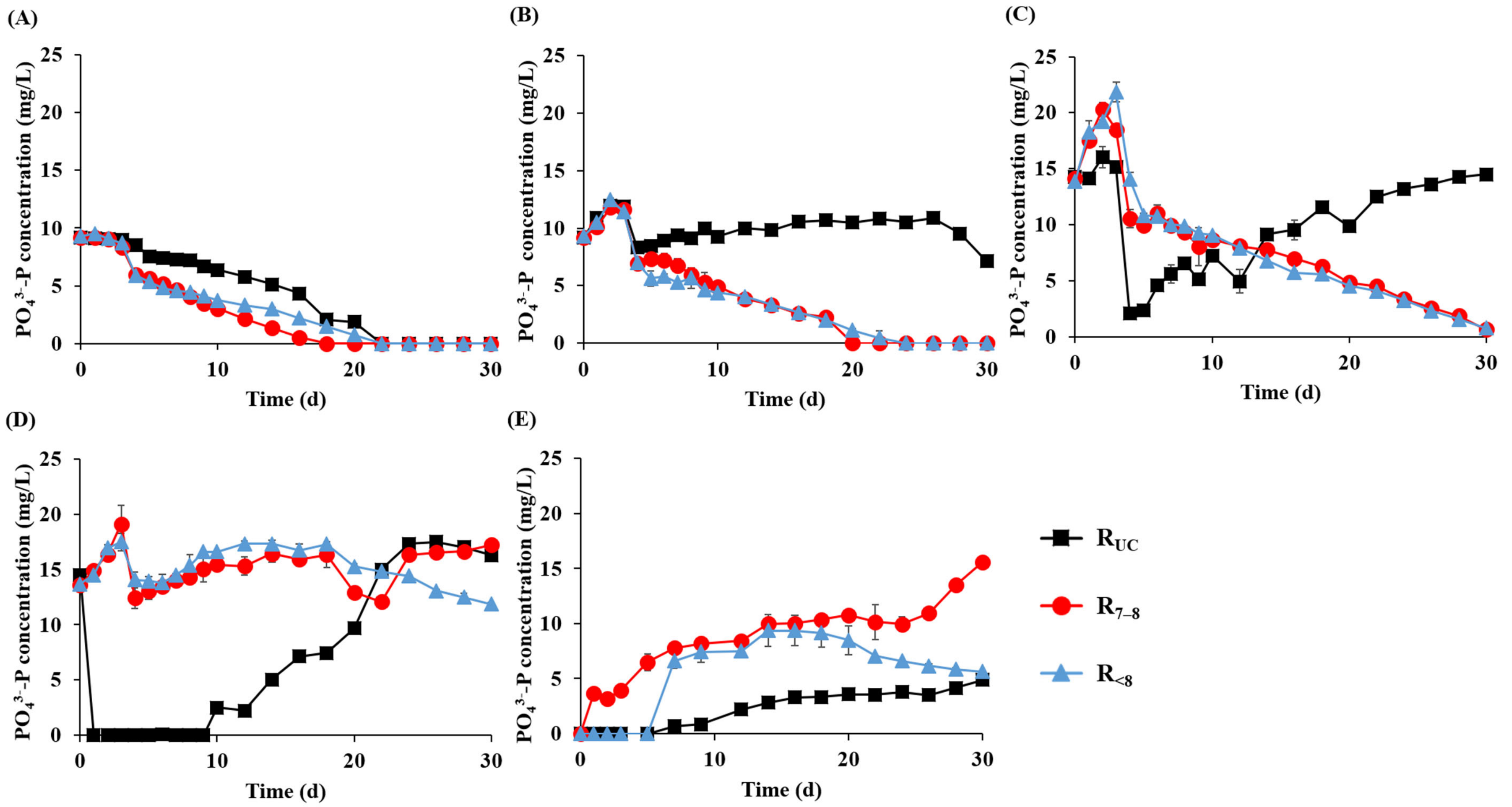

Figure S2. The PO_4^{3-}-P concentration profiles during cultivation of the microalgae cultures with different pH control strategies (R_{UC}, $R_{7–8}$, and $R_{<8}$) at initial NH_4^+-N concentrations of 100, 200, 400, and 800 mg NH_4^+-N/L (A–D) and the uninoculated control cultures (E).

Table S1. Settleability of cultivated biomass.

Culture	12-h settleability (%)	24-h settleability (%)
R_{UC}-100	42.4 ± 1.8	81.8 ± 0.7
$R_{7–8}$-100	23.4 ± 3.5	74.1 ± 0.7
$R_{<8}$-100	93.6 ± 3.1	96.6 ± 0.8
R_{UC}-200	24.6 ± 4.6	59.7 ± 1.4
$R_{7–8}$-200	51.1 ± 0.6	72.0 ± 1.4
$R_{<8}$-200	91.1 ± 0.4	97.3 ± 0.1
R_{UC}-400	n.d.[a]	n.d.
$R_{7–8}$-400	14.8 ± 1.9	68.3 ± 0.9
$R_{<8}$-400	57.9 ± 2.1	93.3 ± 0.3

[a] No visible microalgal growth was observed.

Table S2. Richness and diversity indices of bacterial communities based on the ASV profiles.

Culture or sample	Richness	Shannon	Inverse Simpson
Microalgae inoculum	67	1.364	0.643
Raw AD effluent	350	3.614	0.940
R_{UC}-100	206	3.032	0.898
$R_{7–8}$-100	175	3.173	0.899
$R_{<8}$-100	113	2.904	0.809
R_{UC}-400	225	2.997	0.870
$R_{7–8}$-400	236	3.845	0.962
$R_{<8}$-400	143	1.974	0.652
